# Mammalian Mitophagosome Formation: A Focus on the Early Signals and Steps

**DOI:** 10.3389/fcell.2020.00171

**Published:** 2020-03-18

**Authors:** Maria Zachari, Nicholas T. Ktistakis

**Affiliations:** ^1^MRC Protein Phosphorylation and Ubiquitylation Unit, University of Dundee, Dundee, United Kingdom; ^2^Signalling Programme, Babraham Institute, Cambridge, United Kingdom

**Keywords:** autophagy, mitophagy, mTOR, endoplasmic reticulum, membrane potential

## Abstract

Mitophagy, a conserved intracellular process by which mitochondria are eliminated via the autophagic machinery, is a quality control mechanism which facilitates maintenance of a functional mitochondrial network and cell homeostasis, making it a key process in development and longevity. Mitophagy has been linked to multiple human disorders, especially neurodegenerative diseases where the long-lived neurons are relying on clearance of old/damaged mitochondria to survive. During the past decade, the availability of novel tools to study mitophagy both *in vitro* and *in vivo* has significantly advanced our understanding of the molecular mechanisms governing this fundamental process in normal physiology and in various disease models. We here give an overview of the known mitophagy pathways and how they are induced, with a particular emphasis on the early events governing mitophagosome formation.

## Introduction

Macroautophagy (or simply here autophagy) is a conserved quality control pathway by which a double membrane structure, called an autophagosome, grows to engulf cytoplasmic components in order to deliver them to the lysosome for degradation. Different stimuli can induce either “bulk autophagy” which is a non-selective process degrading random portions of the cytoplasm or “selective autophagy” which is activated to specifically degrade cellular components such as whole organelles (e.g., mitochondria, ER, peroxisomes - to name a few) and protein aggregates (Kirkin, 2019). One of the best understood pathways of selective autophagy is mitophagy - the selective degradation of mitochondria via autophagy. Mitochondria are dynamic organelles whose main function is to produce energy to support the many intracellular processes our cells are constantly undertaking. Mitochondrial quality control is crucial as defects to these organelles can lead to apoptosis or tissue damage, and, unsurprisingly, such mitochondrial defects have been linked to numerous human diseases (Murphy and Hartley, 2018). Thus, elimination of damaged and potentially toxic mitochondria is of extreme importance in favor of homeostasis and survival. A major pathway for the clearance of these mitochondria - and maintenance of mitochondrial network integrity and quality control - is mitophagy. Autophagosomes engulfing mitochondria are called mitophagosomes. Mitophagy occurs in a basal level (with different cell types exhibiting different levels of basal mitophagy) but can also be induced upon stresses such as exercise and ischemia *in vivo* and by mitochondrial disrupting/damaging agents in cultured cells (Montava-Garriga and Ganley, 2019). Impaired mitophagy has been associated with aging and numerous human disorders such as Parkinson’s disease (PD), Alzheimer’s disease (AD), Huntington’s disease (HD), cancer, but also cardiovascular and liver diseases (Palikaras et al., 2017; Um and Yun, 2017; Palikaras et al., 2018). Pharmacological modulation of mitophagy has been suggested to have potential as a therapeutic strategy for the treatment of these diseases. Although there is evidence that mitophagy is involved in pathogenesis, the exact role of mitophagy and mitophagy-related genes in pathological conditions is yet unclear. Ongoing *in vivo* and *in vitro* studies are aiming to elucidate this as well as to explore whether mitophagy could make a good pharmacological target in the context of disease. Over the past two decades, key studies have significantly advanced our understanding of the molecular mechanisms governing mitophagy. Here, we will aim to review the main mitophagy pathways with a particular focus on the early signaling events.

## Autophagy Machinery

The process of forming a double-membrane autophagosome depends on a series of hierarchical steps that bring together more than 30 proteins or protein complexes. Upon inactivation of mTOR (in pathways of non-selective autophagy) the ULK complex composed of the protein kinase ULK1 (or its homolog ULK2), and the adaptors FIP200, ATG13, and ATG101 translocates to endoplasmic reticulum (ER) tubulovesicular membranes that have been “marked” by the presence of ATG9-containing vesicles (Hara et al., 2008; Ganley et al., 2009; Hosokawa et al., 2009b,a; Karanasios et al., 2016). These membranes then recruit the VPS34 complex composed of the PI 3-kinase VPS34 [synthesizing phosphatidylinositol 3-phosphate (PI3P)] and the adaptors VPS15, ATG14, and Beclin-1 which generates PI3P on ER-associated membranes termed omegasomes (Axe et al., 2008). The PI3P-enriched omegasomes then recruit the WIPI effectors and DFCP1, with the former group responsible for bringing on site the lipidation machinery that mediates the covalent modification of ATG8 family members (LC3 and GABARAP families) with phosphatidylethanolamine (PE) (Dooley et al., 2014). These PE-modified ATG8 proteins become part of the autophagosomal membrane whereas all of the other proteins come off as the double membrane closes and travels to the lysosomes for degradation (Axe et al., 2008; Karanasios et al., 2013). One challenge specific to our topic is how this very complicated machinery for making the double membrane autophagosome co-ordinates with the machinery that selects damaged cargo during selective autophagy. We will address this question in later sections.

## Main Mitophagy Triggers *In Vitro* and *In Vivo*

### Induction of Mitophagy *in vivo*

It was recently shown that mitophagy occurs *in vivo* in multiple tissues of mice at steady state without the need of external stimuli. This so-called basal mitophagy occurs presumably to ensure quality control of mitochondria as a housekeeping mechanism (McWilliams et al., 2016; Sun et al., 2017; McWilliams et al., 2018). Apart from its basal occurrence, mitophagy is also induced to support many physiological processes *in vivo* during organismal development. For example, during early embryogenesis, mitophagy has been reported to be responsible for the degradation of paternal mitochondria from the fertilized oocyte and early embryo (Rojansky et al., 2016). Furthermore, during reticulocyte maturation, mitophagy is a key pathway in regulating elimination of mitochondria for the production of mature erythrocytes (Kundu et al., 2008; Sandoval et al., 2008). Mitophagy has been reported to trigger a metabolic switch from oxidative phosphorylation to glycolysis, which is required for retina ganglion cell (RGC) and M1 macrophage differentiation (Esteban-Martinez et al., 2017). Similarly, mitophagy is key in promoting a switch from glycolysis to oxidative phosphorylation in myoblast differentiation (Sin et al., 2016). Apart from its role during embryonic development, mitophagy induced in response to infection has been proposed to have a protective inhibitory effect on the inflammasome, to avoid an excessive immune response which can lead to tissue damage (Kim et al., 2016; Zhong et al., 2016). Multiple physiological stresses have been reported to induce mitophagy in mice, including exercise, starvation, a switch to high fat diet, ischemia and hypoxia. More specifically, acute exercise is a strong mitophagy inducer in heart and skeletal muscle to mediate mitochondrial remodeling (Moyzis et al., 2015; Laker et al., 2017; Drake et al., 2019). Starvation is well known to induce general autophagy in mice, but this stress has also been reported to induce mitophagy, and interestingly there is evidence for canonical and non-canonical mechanisms occurring during starvation-induced mitophagy (discussed below) (Mizushima et al., 2004; Nishida et al., 2009; Hirota et al., 2015; Saito et al., 2019). Cardiomyocytes from mice subjected to high fat diet were shown to exhibit elevated levels of mitophagy to prevent cytotoxicity (Tong et al., 2019), although this resulted in reduced mitophagy in liver (Sun et al., 2015). Myocardial ischemia and energy stress (48 h starvation) have been shown to induce mitophagy in cardiomyocytes of mice, whereas ischemic preconditioning as well as ischemia-reperfusion injury were shown to induce mitophagy in kidney and brain tissues (Tang et al., 2016; Livingston et al., 2019; Saito et al., 2019; Tang et al., 2019). Hypoxic conditions also result in mitophagy induction in multiple tissues in mice and in various cell lines *in vitro* (Zhang et al., 2008; Allen et al., 2013; Sun et al., 2015, Zhang W. et al., 2016).

### Induction of Mitophagy *in vitro*

Apart from the above more physiological ways to trigger mitophagy, chemical inducers of mitophagy have proved to be great tools and have allowed the dissection of the molecular mechanisms of this pathway *in vitro* (mostly in tissue cultured cells). Multiple compounds have been reported to induce mitophagy and have been recently reviewed in detail (Georgakopoulos et al., 2017). For the purpose of this review we will mention a few recent and widely used mitophagy inducers for which some mechanistic detail is known. The mitochondrial uncouplers CCCP and FCCP are proton ionophores which cause severe loss of mitochondrial membrane potential, leading to a robust activation of mitophagy. A combination of the F0F1-ATPase inhibitor oligomycin and the complex III inhibitor antimycin A (symbolized as O/A treatment) also causes membrane depolarization and is known to be a strong mitophagy inducer in cultured cells (Georgakopoulos et al., 2017). The iron chelators depheriprone (DFP) and deferoxamine (DFO) are hypoxia mimicking agents and robustly induce mitophagy in a HIF1α-dependent manner (Allen et al., 2013).

Celastrol, a plant-derived pentacyclic triterpene with reported anti-inflammatory, anti-cancer and anti-obesity effects, was shown to induce mitophagy via Nur77 in a pathway that will be described later on in more detail (Hu et al., 2017). Lastly, we recently reported that the lactone Ivermectin acutely induces a strong mitophagic response upon fragmentation of mitochondria and is the fastest mitophagy inducer to our knowledge (Zachari et al., 2019). The mechanism by which mitophagy is induced by Ivermectin is currently unknown, but it is unlikely that its action is similar to CCCP or FCCP as it reduces oxygen consumption rate (OCR) whereas CCCP and FCCP are well known to dramatically increase OCR (Georgakopoulos et al., 2017; Juarez et al., 2018; Zachari et al., 2019). On the other hand, treatment with Oligomycin/Antimycin A results in decreased OCR as Ivermectin does, although in this case the timings of the two responses are very different with Ivermectin causing a much faster induction of mitophagy. Furthermore, it is unlikely that it acts through a HIF1α-dependent pathway, as this type of mitophagy stimulation requires transcriptional activity and usually takes longer time to occur. Thus, the above compounds appear to act through different mechanisms in inducing mitophagy.

## Mitophagy Signals Before Engaging the Autophagy Machinery: Ubiquitin Dependent and Independent Pathways

**FIGURE 1:**
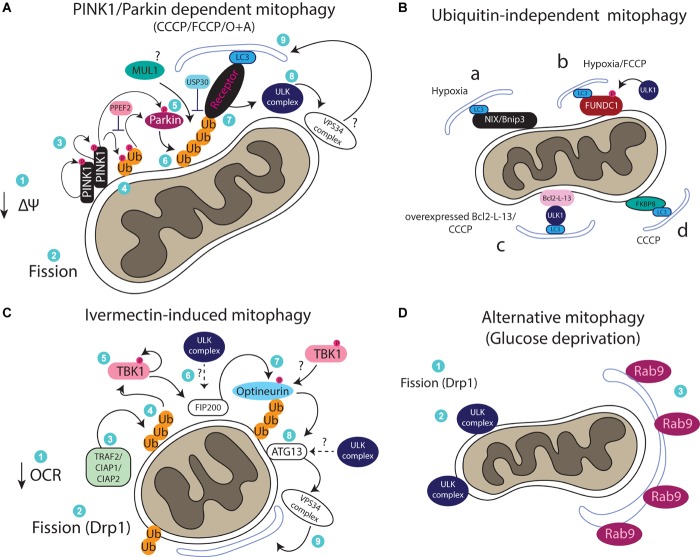
**(A)** Cartoon of the PINK1/Parkin pathway. Upon loss of membrane potential (ΔΨ), PINK1 stabilizes on the OMM, dimerizes and autophosphorylates. PINK1 next phosphorylates Ubiquitin attached onto OMM proteins, leading to the recruitment of Parkin, which also gets phosphorylated and activated by PINK1. Parkin further ubiquitinates OMM proteins leading to the recruitment of receptor proteins for the generation/recruitment of an autophagosome and subsequent degradation of the mitochondrion. **(B)** Representation of mitophagy pathways which do not rely on ubiquitin. Various mitochondrial proteins can act as mitophagy receptors including: (a) NIX/Bnip3, (b) FUNDC1, (c) Bcl2-L-13, and (d) FKBP8. **(C)** Ivermectin-induced mitophagy relies on mitochondrial fragmentation and ubiquitylation via TRAF2/CIAP1/CIAP2. Upon ubiquitylation, TBK1 is required for FIP200 recruitment, which results in optineurin recruitment and downstream activation of ATG13 and the rest of the autophagic machinery for mitophagy. **(D)** An alternative autophagy pathway which does not rely on LC3 lipidation can also mediate mitophagy. This pathway requires mitochondrial fission, ULK1 and Rab9-possitive membranes.

Different pathways can regulate mitophagy and one of the main differences among them is their dependency or not on ubiquitin. The best studied mitophagy pathway is regulated by the Parkinson’s disease related proteins PINK1 and Parkin (Bingol and Sheng, 2016). An illustration of the PINK1/Parkin pathway is shown in Figure 1A. PINK1 is a serine/threonine kinase which contains a mitochondrial-targeting signal. In healthy mitochondria, PINK1 is imported into the mitochondria via the TOM and TIM complexes, following cleavage by mitochondrial proteases including PARL (Jin et al., 2010; Greene et al., 2012). Cleaved PINK1 is exported back to the cytoplasm in an unstable form which is subjected to proteasomal degradation (Yamano and Youle, 2013). Upon mitochondrial depolarization, a loss of mitochondrial membrane potential inhibits import of PINK1 triggering its stabilization on the outer mitochondrial membrane (OMM), which results in its autophosphorylation and dimerization (Okatsu et al., 2012, 2013). PINK1 then regulates E3 ligase Parkin recruitment by phosphorylating ubiquitin at serine 65 attached on multiple OMM proteins (Narendra et al., 2010; Kane et al., 2014; Kazlauskaite et al., 2014). Phosphorylated ubiquitin acts as a key signal for the recruitment and activation of Parkin (Wauer et al., 2015). Parkin activation also requires direct phosphorylation by PINK1 at serine 65 (Kondapalli et al., 2012; Shiba-Fukushima et al., 2012). Parkin then further conjugates ubiquitin on OMM proteins, marking the mitochondria for degradation by the autophagic machinery (Narendra et al., 2008). It is worth mentioning here that phosphorylation of ubiquitin by PINK1 has been reported to be sufficient to recruit mitophagy cargo receptor proteins (primarily optineurin and NDP52) independently of Parkin but this is enhanced by Parkin activity (Lazarou et al., 2015). Recent work revealed that the phosphatase PPEF2 is responsible for dephosphorylating ubiquitin at serine 65 to oppose the PINK1 effect and inhibit mitophagy (Wall et al., 2019). The deubiquitinating enzyme that reverses this process by removing ubiquitin from the OMM is USP30 (Bingol et al., 2014). Another E3 ligase that has been reported to act in parallel with Parkin, is MUL1 (Yun et al., 2014; Rojansky et al., 2016). The upstream signals that regulate MUL1 recruitment and activity require further study, as the process has been reported to be both dependent and also independent of PINK1, raising the possibility of context dependent regulation of this protein (Yun et al., 2014; Rojansky et al., 2016). Multiple other proteins have been reported to regulate PINK1/Parkin-mediated mitophagy. More specifically, choline dehydrogenase (CHCD), a mitochondrial enzyme regulating methionine synthesis, was shown to act as a ubiquitin “eat me” signal and to associate with p62 for phagophore recruitment (Park et al., 2014). Depletion of CHCD was reported to impair PINK1/Parkin mitophagy induced by CCCP (Park et al., 2014). Similarly, two mitochondrial matrix proteins required for PINK1/Parkin mitophagy, NIPSNAP1 and NIPSNAP2, were recently reported to accumulate on mitochondria upon CCCP or hypoxia treatment, to act as “eat me” signals by directly recruiting mitophagy adaptor proteins (NDP52 and p62) as well as LC3/GABARAPs (Princely Abudu et al., 2019). Apart from receptor proteins, cardiolipin, a phospholipid normally localized in the inner mitochondrial membrane (IMM), translocates to the OMM upon Rotenone (a complex I inhibitor) and FCCP/CCCP treatment to act as a receptor for LC3 and thus mediate autophagosomal engulfment of damaged mitochondria (Chu et al., 2013). Cardiolipin potentially acts downstream of PINK1/Parkin signaling, although further research is required to confirm this scenario (Chu et al., 2013). An inner membrane mitochondrial protein, prohibitin 2, was also reported to mediate mitophagy by binding to LC3 upon proteasomal-dependent OMM rupture caused by O/A treatment (Wei et al., 2017). The presence of prohibitin 2 is important for PINK1/Parkin mitophagy but also for elimination of paternal mitochondria upon oocyte fertilization in *C. elegans* (Wei et al., 2017). It was recently suggested that prohibitin 2 might be involved in PINK1/Parkin mitophagy by positively regulating PINK1 stabilization onto mitochondria (Yan et al., 2019).

We recently reported a second ubiquitin-dependent mitophagy pathway which is rapidly induced by the lactone Ivermectin (Zachari et al., 2019). Ivermectin treatment results in acute mitochondrial damage as observed by a decrease in OCR and severe fragmentation of the mitochondrial network. This leads to induction of mitophagy, independently of PINK1 and Parkin, but dependent on the E3 ligases TRAF2, CIAP1, and CIAP2 which work synergistically (and potentially in complex) to conjugate ubiquitin onto fragmented mitochondria. Upon ubiquitination, TBK1 becomes activated in order to regulate recruitment of the adaptor protein optineurin, to mediate mitophagy in a pathway which will be discussed later in more detail (Zachari et al., 2019) (Figure 1C). The DUB enzyme(s) reversing this process is yet to be identified. Apart from optineurin and NDP52, several other adaptor proteins are involved in mitophagy of ubiquitinated mitochondria including TAX1BP, p62, and NBR1 (Geisler et al., 2010; Moore and Holzbaur, 2016; Zachari et al., 2019). Another TRAF2/ubiquitin-dependent mitophagy pathway occurs in the presence of celastrol and the cytokine TNFa (Hu et al., 2017). More specifically, under these conditions, celastrol binds to Nur77 (a transcription factor nuclear receptor protein which can translocate to mitochondria to induce apoptosis), resulting in its translocation to mitochondria. When on mitochondria, Nur77 recruits TRAF2 resulting in mitochondrial ubiquitination and p62 recruitment for autophagic elimination in an anti-inflammatory mechanism (Hu et al., 2017).

Mitophagy can also occur independently of the presence of ubiquitin on the mitochondria (Figure 1B). These mitophagy pathways are regulated by adaptor proteins containing mitochondrial-targeting domains and LC3-interacting region (LIR) motifs. Whereas only one yeast mitophagy receptor has been identified so far (the protein ATG32) (Kanki et al., 2009), multiple mitochondrial receptors have been identified in mammals, namely: BNIP3, NIX (or BNIP3L), FUNDC1, and Bcl2-L-13. BNIP3 and NIX are two very similar proteins with pro-apoptotic functions that are transcriptionally upregulated upon hypoxia in a HIF1α-dependent manner to mediate hypoxia-induced mitophagy (Zhang et al., 2008; Hanna et al., 2012, Ney, 2015; Yuan et al., 2017). NIX is also required for mitophagy to mediate mitochondrial removal during reticulocyte maturation (Schweers et al., 2007) and was recently reported to mediate ischemia-reperfusion-induced mitophagy in the brain (Yuan et al., 2017). Furthermore, NIX overexpression can rescue mitophagy induced upon CCCP treatment in cells deficient for Parkin-mediated mitophagy (Koentjoro et al., 2017). The interaction of NIX with LC3 during mitophagy has been suggested to be regulated by phosphorylation of NIX (at serine residues 34 and 35), although the kinase mediating these events is not yet identified (Rogov et al., 2017). Furthermore, NIX was recently reported to be a substrate of Parkin during mitophagy (Gao et al., 2015). Parkin-mediated ubiquitination of NIX results in the recruitment of NBR1 to mitochondria to mediate their autophagosomal engulfment and removal (Gao et al., 2015). Interestingly, BNIP3 has been reported to promote PINK1-dependent mitophagy as well, by stabilizing PINK1 levels onto mitochondria in hypoxic conditions (Zhang T. et al., 2016). Of note, overexpression of BNIP3 alone causes membrane potential loss and is sufficient to induce mitophagy, highlighting its significant role in activating this pathway (Rikka et al., 2011). The OMM protein FUNDC1 has been reported to regulate mitophagy induced upon hypoxia, but also upon mitochondrial depolarization with FCCP (Liu et al., 2012; Chen et al., 2014). Basal phosphorylation of FUNDC1 by Src (at tyrosine 18) and CK2 (at serine 13) inhibit its interaction with LC3 and concomitantly mitophagy, whereas hypoxia-induced dephosphorylation increases its interaction with LC3 to promote mitophagy (Liu et al., 2012; Chen et al., 2014). FUNDC1 has also been reported to be regulated by direct phosphorylation by ULK1 (at serine 17) to promote mitophagy both in the context of hypoxia and of FCCP treatment (Wu et al., 2014). Recently, NIX and FUNDC1 have been reported to be essential for mitophagy during cardiac progenitor cell differentiation, mediating mitochondrial network remodeling (Lampert et al., 2019). Bcl2-L-13 has been suggested to be the mammalian homolog of the yeast Atg32 due to sequence similarity and because it can rescue mitophagy in yeast upon loss of Atg32 (Murakawa et al., 2015). Bcl2-L-13 is thought to regulate mitophagy both by binding to LC3 but also by mediating mitochondrial fission (Murakawa et al., 2015). Another recently discovered mitophagy adaptor, FKBP8 with a preferred binding to LC3A, induces mitochondrial fragmentation and mitophagy when overexpressed in cells, independently of the PINK1/Parkin pathway (Bhujabal et al., 2017) (Figure 1B).

## Recruitment of the Autophagic Machinery

Since receptor proteins are central in mediating mitophagy and can bind both mitochondria and LC3/GABARAP proteins, it was thought until recently that they mediate recruitment of the autophagic machinery by binding to LC3/GABARAP-positive forming phagophores. Recent evidence suggests that both ubiquitin dependent and independent mitophagy adaptor/receptor proteins can also act as hubs for the recruitment of proteins involved in early autophagy events and working upstream of LC3/GABARAPs such as ULK1 complex components and the PI3P-binding proteins WIPIs and DFCP1. These data reveal that mitophagy can occur not only by utilizing already forming autophagosomes but also by direct initiation of autophagosome formation via activation of early components. A study by Itakura et al. showed that during Parkin-mediated mitophagy, early autophagy proteins such as ULK1, ATG14, DFCP1, WIPI1, and ATG16L1 translocated onto damaged mitochondria even in the absence of LC3 conjugation on membranes (Itakura et al., 2012). It was later shown by the Youle laboratory that this is mediated in a receptor-dependent manner (Lazarou et al., 2015). More specifically, upon mitochondria depolarization, phospho-ubiquitin produced as a result of PINK1 activation, recruits the receptor proteins NDP52 and optineurin, which are required for the recruitment of ULK1 and the omegasome markers WIPI1 and DFCP1 (Lazarou et al., 2015). Interestingly, later work showed that ectopic targeting of NDP52 onto mitochondria is sufficient to recruit the ULK1 complex (via a direct interaction of NDP52 with FIP200), ATG14 and ATG16L1 and concomitantly induce mitophagy in both Parkin-dependent and independent mechanisms (Vargas et al., 2019). In the same study it was shown that targeting of ULK1 to mitochondria was sufficient to induce mitophagy (even when NDP52 or TBK1 are absent), supporting the notion that the function of early ubiquitin/receptor signals is to recruit the autophagy initiation machinery for the *de novo* formation of an autophagosome on the target mitochondrion. The key event regulated by NDP52-mediated recruitment of the ULK1 complex is a direct interaction between NDP52 and FIP200 which requires the presence and activation of TBK1 (Vargas et al., 2019). Binding of receptor proteins to FIP200 is not a mitophagy specific mechanism. It was recently reported to be the key event in other types of selective autophagy as well including ER-phagy, aggrephagy, xenophagy, and pexophagy (Smith et al., 2018; Ravenhill et al., 2019; Turco et al., 2019) highlighting that different selective autophagy pathways share common features. In addition, it was recently shown that LC3/GABARAP proteins can mediate recruitment of the adaptor proteins optineurin and NDP52 to mitochondria, in a positive feedback loop in order to accelerate PINK1/Parkin mediated mitophagy (Padman et al., 2019). Interestingly, LC3/GABARAPs likely play different roles during PINK1/Parkin mitophagy as GABARAPs appear to be more essential than the LC3s (Nguyen et al., 2016). Furthermore, loss of all LC3/GABARAPs did not abolish autophagosome formation (as seen by EM and recruitment of earlier autophagy markers such as ULK1 on mitochondria) but it rather caused defects in mitophagosome-lysosome fusion (Nguyen et al., 2016). Independently of PINK1 and Parkin, we recently showed that ubiquitination of fragmented mitochondria induced by Ivermectin leads to activation of TBK1 which is required for optineurin recruitment and mitophagy (Zachari et al., 2019). In this mitophagy pathway, recruitment of optineurin required FIP200 but not ULK1/2 or ATG13, revealing that the activity of the ULK1/2 kinases is not essential for certain mitophagy pathways. [It is worth mentioning here that the FIP200 and ATG13 proteins have been previously reported to act independently of the complex during autophagy (Alers et al., 2011) although the exact mechanisms underlying these functions are poorly understood.] Furthermore, TBK1 activation was hierarchically earlier than FIP200, and was required for optineurin recruitment. Given the recently published evidence discussed above, it is possible that a direct interaction between optineurin (and possibly more adaptors) and FIP200 is key also for the progression of Ivermectin-induced mitophagy. ATG13 was required for the formation of the omegasomes, within which the LC3-positive mitophagosomes formed (Zachari et al., 2019). ATG9 vesicles - essential components of the autophagic machinery - have been involved in both PINK1/Parkin and Ivermectin-induced mitophagy, although their exact role during mitophagy remains elusive (Itakura et al., 2012; Yamano et al., 2018, Zachari et al., 2019).

Mitophagy induced via Bcl2-L-13 was also recently reported to involve recruitment of ULK1 to mitochondria, even though this was shown to occur in an indirect manner whereby Bcl2-L-13 recruits LC3B, leading to recruitment of ULK1 which binds to LC3B via a LIR motif (Murakawa et al., 2019). As mentioned earlier, FUNDC1-dependent mitophagy also involves recruitment of ULK1 to the mitochondria. In this case FUNDC1 is phosphorylated by ULK1 which causes enhanced binding to LC3 during mitophagy (Wu et al., 2014). Predictably, ULK1 kinase activity is required for this pathway (Wu et al., 2014) (Figure 1B).

Even though mitophagy generally is reported to occur via the canonical autophagic machinery that we described earlier, a non-canonical mitophagy pathway which does not require LC3 lipidation was reported to occur in cardiomyocytes upon ischemic stress and starvation as well as in HeLa cells upon starvation and hypoxia (Hirota et al., 2015; Saito et al., 2019). In cardiomyocytes, this pathway depends on ULK1 but not on ATG5 and does not involve LC3-positive autophagosomes; instead it is mediated by Rab9 positive membranes/vesicles which drive lysosomal degradation of mitochondria. It is currently unknown whether ubiquitin signals and receptor proteins are involved in ULK1-Rab9 dependent mitophagy (Saito et al., 2019) (Figure 1D). Rab9-mediated autophagy has been previously reported as a non-canonical/alternative non-selective autophagy pathway in cells lacking ATG5, where it was also suggested that it plays a role in mitochondrial clearance during erythrocyte differentiation (Nishida et al., 2009).

**FIGURE 2:**
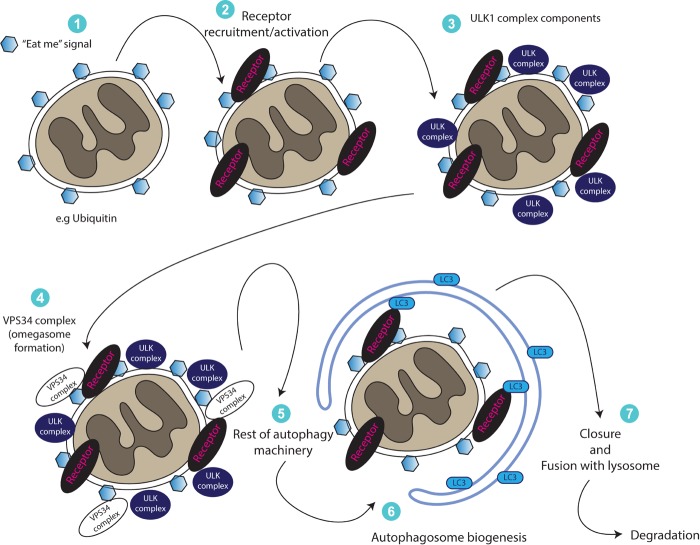
A generalized illustration of mitophagy. Upon damage or other stimulus, mitochondria become positive for molecules resulting in an “eat me” signal. This results in recruitment (or activation of already existing) receptor proteins, which regulate the recruitment of forming autophagosomes or that of early autophagy machinery proteins (ULK complex, VPS34 complex) for the generation of a double membrane mitophagosome. Ultimately, mitophagosomes fuse with the lysosomes to degrade themselves.

It is important to mention here that mitochondrial fission (the separation from the network or fragmentation), appears to be an essential (but not sufficient) step for mitophagy (Twig et al., 2008; Williams and Ding, 2018). A key regulator of mitochondrial fission is Drp1 - a member of the dynamin family of large GTPases (Labrousse et al., 1999). Although multiple reports have shown that Drp1 is required for both PINK1-dependent and independent mitophagy pathways (Lee et al., 2011; Kageyama et al., 2014; Wu et al., 2016; Li et al., 2019; Zachari et al., 2019), other studies suggest that it might be dispensable for mitophagy (Murakawa et al., 2015; Yamashita et al., 2016; Burman et al., 2017). Thus, mitophagy dependency on Drp1 could be potentially context specific and further investigation is needed to understand its role in this pathway. A generalized illustration of our current understanding of mitophagy signaling is shown in Figure 2.

## Does mTORC1 Regulate Mitophagy?

As mentioned earlier, one of the master regulators of bulk autophagy is mTORC1, which when active, suppresses autophagy via direct phosphorylation of the ULK complex components ULK1 and ATG13 (Ganley et al., 2009; Hosokawa et al., 2009a; Kim et al., 2011; Puente et al., 2016). Even though mTORC1 has a well-established role during starvation-induced autophagy, its role during mitophagy is unclear. Inhibition of mTORC1 with rapamycin has been reported to improve mitochondrial health (Nacarelli et al., 2018). Studies with CCCP and FCCP in a non-mitophagy context, have reported that these proton uncouplers cause a reduction in mTORC1 activity, suggesting that PINK1/Parkin mitophagy might require mTOR inactivation as bulk autophagy does (Inoki et al., 2003; Kim et al., 2013; Bartolome et al., 2017). Furthermore, recent reports suggest that mTORC1 hyperactivation can have an inhibitory effect on PINK1/Parkin mediated mitophagy as well as general autophagy (Bartolome et al., 2014, 2017; Bordi et al., 2019). Importantly, hypoxia leads to a reduction in mTORC1 activity as well, meaning that mTORC1 might play a role in hypoxia-induced mitophagy (Vadysirisack and Ellisen, 2012). Ivermectin did not appear to affect mTORC1 activity in the time points and concentrations we used to induce mitophagy (although treatments of 24–48 h have been shown to have an inhibitory effect on mTORC1) (Dou et al., 2016; Liu et al., 2019; Zachari et al., 2019). This might mean that the ULK complex can mediate mitophagosome formation even if it is phosphorylated by mTORC1 or that mitophagy is mediated by a pool of ULK complex that has escaped inhibition of mTORC1. Vargas et al. (2019), showed that mitophagy-induced due to ULK1 targeting to mitochondria was unaffected by mTOR overexpression and concomitant increase in ULK1 phosphorylation. It is important to mention here that amino acid (or full nutrient) starvation has been shown to block mitophagy in multiple cell lines as a result of reduced mitochondrial fission, even though mTORC1 activity is lost in these conditions (Gomes et al., 2011; Rambold et al., 2011). Thus, mTORC1 inactivation does not necessarily lead to mitophagy induction. Most of the mitophagy studies do not evaluate mTORC1 activity and our knowledge on this is limited. In conclusion further research is required to understand its involvement during mitophagy. Of note, AMPK appears to play an important (but not fully understood) role during mitophagy in multiple contexts and it is worth mentioning that mTORC1 regulates AMPK and that both these kinases can potentially influence mitophagy via ULK1. The literature on this topic has recently been reviewed (Herzig and Shaw, 2018).

## Is There a Membrane Source Specific for Mitophagy?

The origin of the autophagosomal membrane during bulk autophagy induced by amino acid starvation is a long-standing question: autophagosomes form *de novo* and the membranes contributing to autophagosomal growth are devoid of protein-markers of the donor membranous compartments, making it hard to identify their provenance. Different organelles and membrane compartments have been proposed to contribute membrane to the autophagosome forming upon amino acid starvation, and these include the ER, Golgi, plasma membrane, mitochondria, and endosomes (Ktistakis and Tooze, 2016; Wei et al., 2018). As discussed earlier, during amino acid starvation autophagosomes form in ER platforms called omegasomes which are marked by the omegasome marker DFCP1 (Axe et al., 2008). Thus, a candidate membrane donor has been proposed to be the ER, with contributions from virtually all intracellular membranes in a way not entirely clear (Ktistakis, 2020). When it comes to mitophagy and other types of selective autophagy our understanding of the membrane source(s), and whether this differs from bulk autophagy, is even less clear. For Ivermectin-induced mitophagy we recently showed that at an early step of the process mitochondria became entrapped within ER strands prior to omegasome formation and mitophagosome generation (Zachari et al., 2019). In our analysis by both live imaging and electron tomography it appeared that the autophagosomal membrane engulfing the mitochondria grew as an extension of the neighboring ER strands-such close apposition of ER with targeted mitochondria was evident in some older publications studying PINK1/Parkin-induced mitophagy (discussed in Zachari et al., 2019). Since observations from other research groups support the notion that mitophagosomes (independently of induction mode) form within omegasomes (Yang and Yang, 2013; Wong and Holzbaur, 2014; Gelmetti et al., 2017; Hsieh and Yang, 2019) it is possible that the ER plays a key role as a membrane source for mitophagy in general. It will be interesting to determine if the different ER proteins that have been reported to be involved in autophagosome biogenesis upon amino acid starvation are also important for mitophagy as well (Walker and Ktistakis, 2019; Ktistakis, 2020). It was recently reported that in yeast mitochondria-ER contact sites are crucial for mitophagy (and pexophagy) to occur (Bockler and Westermann, 2014; Liu et al., 2018). Given the importance of ER-mitochondrial contacts in autophagosome formation in general, it is likely that they are crucial for mitophagy in mammals too, although this is yet to be confirmed experimentally (Hamasaki et al., 2013).

## Conclusion and Future Perspectives

Our understanding of mitophagy signaling process and function has significantly advanced in the past decade. It is now clear that multiple proteins are involved in the regulation of mitophagy and various physiological events or types of stress can induce different mitophagy pathways both *in vivo* and *in vitro*. The PINK1/Parkin pathway has attracted a lot of attention, partially due to the importance of these proteins in Parkinson’s disease and undoubtedly as a result of the detailed mechanistic studies that have dissected it. However, it is now evident that this pathway is not required for the regulation of basal mitophagy (as well as other types of mitophagy) in mice and it might be more relevant in clearing mitochondria following particular damage/stress. Thus, more work is required to understand its physiological relevance (McWilliams et al., 2018; Drake et al., 2019). In terms of other types of mitophagy, there are still a lot of unanswered questions remaining in the field. For example, what kind of damage exactly do the mitophagy-inducing agents cause to the mitochondria? How can this relate to mitochondrial stress in humans (e.g., exposure to environmental hazards or aging)? How can this lead to disease? Can mitophagy be targeted for the development of therapeutics? Related to the last question, biochemical advances in the autophagy field, led to the generation of therapeutically promising compounds called “AUTACs” which can bind to mitochondria causing their ubiquitylation and induction of mitophagy (Takahashi et al., 2019). This way, AUTACs were shown to successfully drive mitochondria to the autophagosomal lumen for degradation, opening up an exciting new era in preclinical research on selective autophagy and its potential in treatment development.

## Author Contributions

MZ and NK wrote and edited the manuscript.

## Conflict of Interest

The authors declare that the research was conducted in the absence of any commercial or financial relationships that could be construed as a potential conflict of interest.
